# Diagnostic performance with sensitivity/specificity optimized computer-aided detection to detect common abnormalities in chest radiography

**DOI:** 10.1007/s11604-026-02053-y

**Published:** 2026-08-03

**Authors:** Miyuki Tomiyama, Masahiro Yanagawa, Tsubasa Yoshida, Kuniaki Ohara, Chiaki Masuda, Keisuke Ninomiya, Shuhei Doi, Atsushi Nakamoto, Masatoshi Hori, Masahiro Hashimoto, Noriyuki Tomiyama

**Affiliations:** 1 Artificial Intelligence in Diagnostic Radiology, The University of Osaka Graduate School of Medicine, Suita, Japan; 2 Department of Radiology, Keio University Graduate School of Medicine, Tokyo, Japan; 3 Department of Diagnostic and Interventional Radiology, Graduate School of Medicine, The University of Osaka, 2-2 Yamadaoka, Suita, 5650871 Osaka Japan

**Keywords:** Chest radiography, Computer-aided detection, Lung nodules, Artificial intelligence, Pneumothorax

## Abstract

**Purpose:**

To evaluate how two fixed computer-aided detection (CAD) operating modes—sensitivity-optimized (SE-CAD) and specificity-optimized (SP-CAD)—affect radiologists’ diagnostic performance in chest radiography (CXR). Previous studies have focused on the performance of standalone CAD at different threshold settings. However, in clinical practice, CAD is commonly used as a decision-support tool for radiologists. This study aimed to evaluate the impact of different CAD threshold settings on radiologists’ diagnostic performance.

**Materials and methods:**

This retrospective single-center diagnostic accuracy study evaluated posteroanterior or anteroposterior CXRs acquired between 2013 and 2023, interpreted with and without AI assistance, using CT as the reference standard. Six radiologists (three residents / three board-certified) read all cases four times over two sessions: first without CAD, then with either SE_CAD or SP_CAD, and after 1-month washout period, second session using the alternative CAD.

**Results:**

375 patients (mean age, 65 ± 15 years; 170 women) were included. The dataset comprised 139 cases with pulmonary opacities (nodules / masses / consolidations), 119 with pleural effusion, 29 with pneumothorax, and 161 normal cases, including overlaps. SP_CAD-assisted readings showed higher specificity than SE_CAD-assisted for pulmonary opacities (*p* < 0.001). Sensitivity did not differ significantly between SE_CAD and SP_CAD assistance. For pulmonary opacities, CAD-assisted reading showed higher sensitivity (both CAD: *p* < 0.001) but lower specificity (SE_CAD: *p* = 0.006; SP_CAD: *p* = 0.03) than unassisted reading. For pleural effusion, CAD-assisted improved sensitivity (SE_CAD: *p* < 0.001; SP_CAD: *p* = 0.02) and specificity (both CAD: *p* < 0.001) than unassisted reading. For pneumothorax, SE_CAD assistance did not significantly affect sensitivity but decreased specificity, whereas SP_CAD assistance improved sensitivity without significantly affecting specificity.

**Conclusion:**

The specificity-prioritized CAD showed higher specificity than the sensitivity-prioritized CAD, without a significant reduction in sensitivity. These findings suggest that different CAD operating points can influence radiologists’ diagnostic performance.

**Secondary abstract:**

We compared radiologist performance using sensitivity-optimized and specificity-optimized computer-aided detection (CAD). Different predefined CAD operating points influenced radiologist performance, resulting in differences in specificity for nodules/masses/consolidations. Our results show that different preset decision thresholds within the same AI model can influence diagnostic performance in AI-assisted radiograph interpretation.

**Supplementary Information:**

The online version contains supplementary material available at https://doi.org/10.1007/s11604-026-02053-y.

## Introduction

Chest radiographies (CXRs) are widely used in clinical practice due to their rapid acquisition, low cost, and effectiveness for screening conditions such as pneumonia and lung cancer. Recently, artificial intelligence (AI)-based systems have attracted attention for their potential to improve diagnostic accuracy and reduce the workload of medical professionals. Several studies have shown that AI assistance can enhance physicians’ ability to detect thoracic abnormalities on CXRs and reduce diagnostic errors [1–3]. Furthermore, computer-aided detection (CAD) systems can improve workflow efficiency and help to alleviate radiologists’ burden [4–6].

In clinical practice, achieving an appropriate balance between sensitivity and specificity is essential, as low specificity may lead to unnecessary follow-up examinations and overdiagnosis, whereas low sensitivity may result in missed diagnoses, both of which have important clinical implications. Although several studies have explored different CAD threshold settings, these evaluations have been limited to standalone CAD performance [7–11]. In clinical practice, particularly in Japan, CAD is rarely used in isolation and instead serves as a decision-support tool for radiologists. Therefore, this study aimed to evaluate how different CAD threshold settings influence radiologists’ diagnostic performance. We hypothesized that fixed, pre-specified CAD operating modes [sensitivity-optimized (SE_CAD) and specificity-optimized (SP_CAD), not user-adjustable] would differentially affect radiologists’ diagnostic performance in detecting pulmonary opacities (nodules/masses/consolidations), pleural effusion, and pneumothorax.

## Materials and methods

This retrospective study received institutional review board approval from our institution’s ethics review board, with a waiver of informed consent. Patient-identifying information was replaced with fictitious data or removed before processing. The study followed Standards for Reporting of Diagnostic Accuracy Studies (STARD) guidelines [12]. Technical support was provided by X Corporation, which had no role in study design, data collection, analysis, or manuscript preparation. The corresponding author had complete control of data and submitted information.

### Study participants

**Fig. 1:**
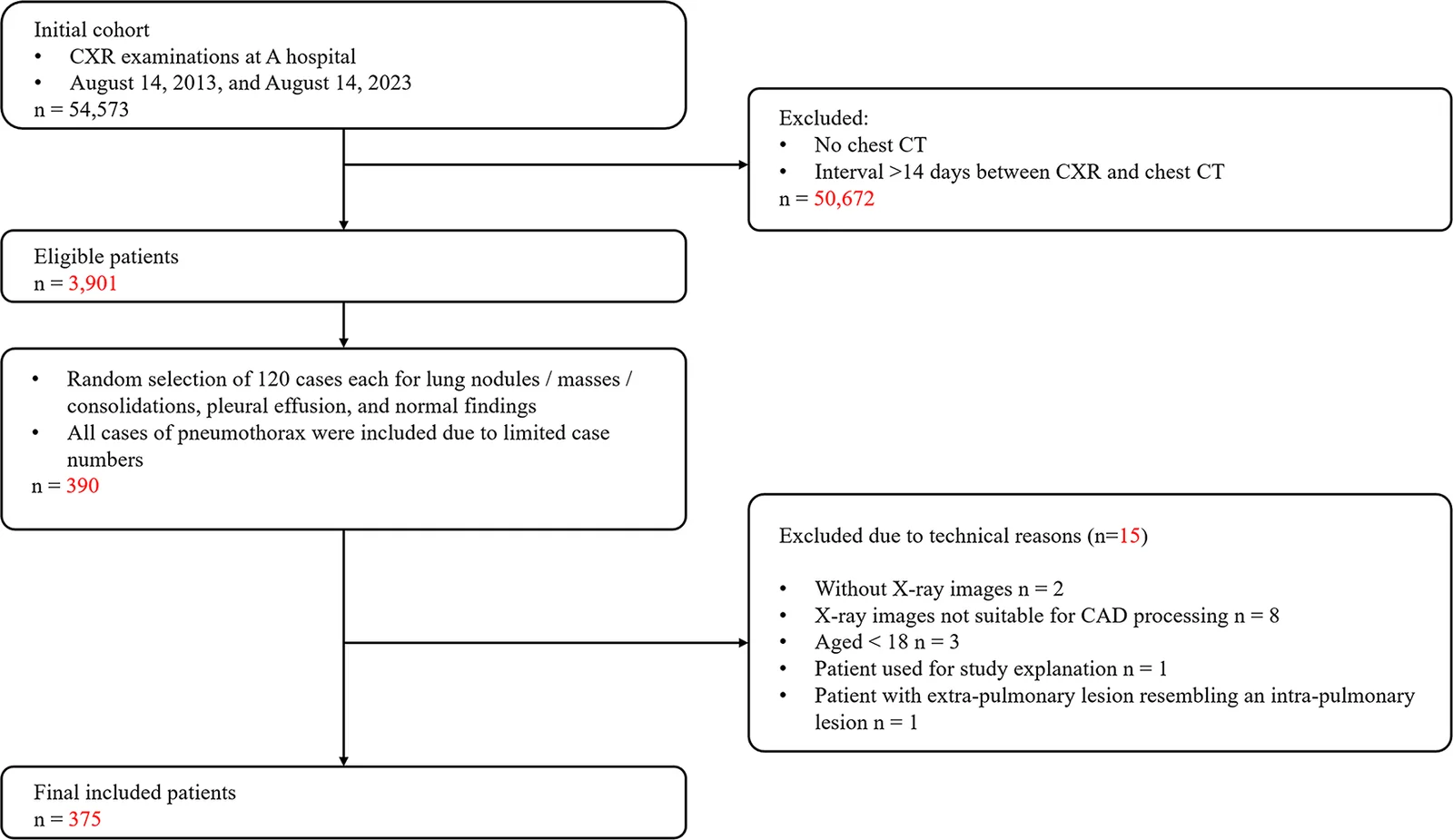
Flow chart showing patient selection criteria for the study cohort. A total of 375 patients were included. CXR = chest radiography

The patient selection process is illustrated in Fig. 1. From 54,573 patients who underwent CXRs at the A hospital between August 14, 2013, and August 14, 2023, investigators (6 and 25 years of experience) identified 3,901 eligible patients based on availability of both CXR and chest CT within 14 days. From eligible patients, 390 cases were selected, including a random sample of 120 cases each for pulmonary opacities, pleural effusion, and normal findings. All pneumothorax cases (*n* = 29) were included due to the limited number of available cases. Patients could have multiple abnormalities. Exclusions (*n* = 15) included: no CXR images (*n* = 2), poor image quality (*n* = 8), age under 18 years (*n* = 3), reader training case (*n* = 1), and extra-pulmonary lesion mimicking intra-pulmonary abnormality (*n* = 1). Finally, a total of 375 patients were included. Table 1 shows the characteristics of patients. The mean age was 65.4 ± 14.8 years and 170 of 375 patients (45%) were women. The dataset comprised 139 cases with pulmonary opacities, 119 with pleural effusion, 29 with pneumothorax, and 161 normal cases, including overlaps. For the stratified analysis of pulmonary opacities, 85 cases were classified as nodules (< 3 cm), 16 as masses (≥ 3 cm), and 38 as consolidations. For pleural effusions, 51 cases were small, 55 cases were moderate, and 7 cases were large. Pneumothoraxes were classified as mild (*n* = 6), moderate (*n* = 17), and severe (*n* = 5). CXRs contained visible medical devices, including pacemakers (*n* = 17), central venous catheters (*n* = 9), ports (*n* = 5), chest tubes (*n* = 2), nasogastric tubes (*n* = 3), gastrostomy (*n* = 2), and aortic stent graft (*n* = 1), with some cases overlapping. CXRs were identified through picture archiving and communication system (PACS), integrated with the electronic medical record system (MegaOakHR), and anonymized.

**Table 1:** Characteristics of Patients

				Value
Characteristic	Age (years)			65.4 ± 14.8
	Sex (female)			170 (45)
Radiographic projection	PA			363 (97)
	AP			12 (3)
Findings	Abnormal			214 (57)
	Nodules, Masses and Consolidations			139
	PA + AP		Nodules	85
			Masses	16
			Consolidations	38
	Pleural effusion			119
	PA		Small	51
			Moderate	55
			Large	7
	Pneumothorax			29
	PA		Mild	6
			Moderate	17
			Severe	5
	Normal			161 (43)
Device	With			37
	Without			338

Data are presented as number of patients, with percentages in parentheses for categorical variables. Age is reported as mean ± standard deviation. Radiographic projections included PA and AP views. Abnormal findings included nodules / masses / consolidations (pulmonary opacities), pleural effusion, and pneumothorax. Some patients had multiple abnormal findings. A total of 214 patients (57%) had at least one abnormal finding, while 161 patients (43%) had normal radiographs (some cases overlapped in categories)

Pulmonary opacities were categorized as nodules (< 3 cm), masses (≥ 3 cm), or consolidations. Pleural effusions were classified as small, moderate, or large, and pneumothoraxes as mild, moderate, or severe. AP radiographs were excluded from the stratified analyses of pleural effusion and pneumothorax because severity assessment is difficult on AP images. Cases were also stratified according to the presence or absence of medical devices. Definitions and details are described in Supplementary 6

PA = posteroanterior; AP = anteroposterior

### Target findings

**Fig. 2:**
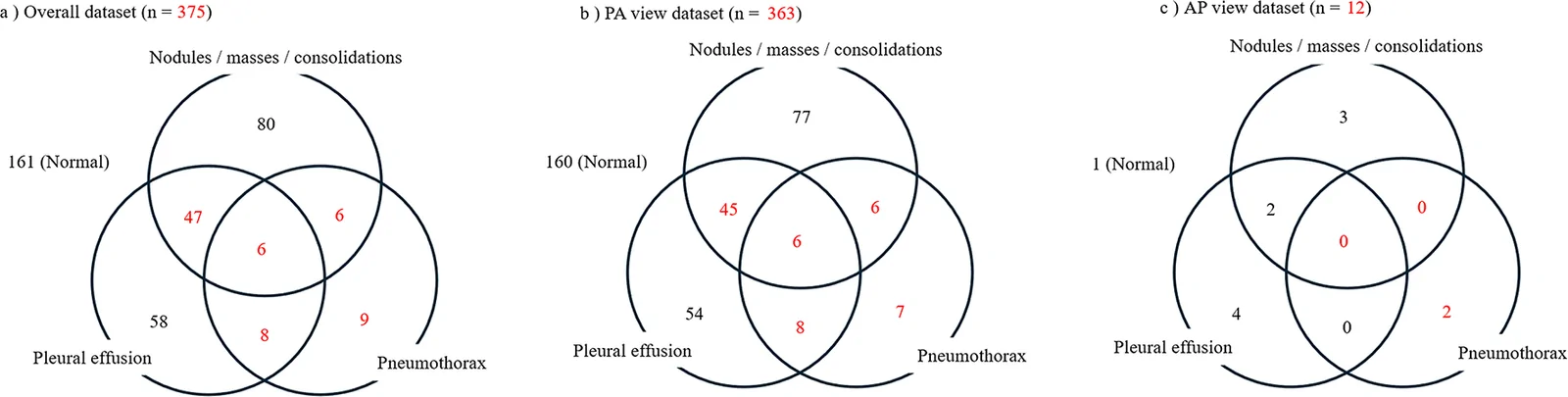
Breakdown of the 375 final selected cases by radiographic projection and diagnostic category (numbers indicate cases). PA views accounted for 363 cases, while AP views comprised 12 cases. The diagnostic distribution included 139 cases with nodules, masses, or consolidations; 119 with pleural effusion; 29 with pneumothorax; and 161 normal cases (some cases overlapped in categories). PA = Posteroanterior, AP = anteroposterior

This study evaluated three thoracic abnormalities: pulmonary opacities (nodules/masses/consolidations), pleural effusion, and pneumothorax. Among 375 cases, 139 were labeled as pulmonary opacities, 119 as pleural effusion, 29 as pneumothorax, and 161 as normal (Table 1; Fig. 2). Some cases overlapped in categories, as patients could present with multiple abnormalities simultaneously (Fig. 2). Both posteroanterior (PA) and anteroposterior (AP) CXRs were included; 12 were AP views. For the stratified analysis of pulmonary opacities, a single finding was assigned to each case (Table 1). When multiple nodules or masses were present, the largest lesion was selected. If consolidation coexisted with nodules or masses, nodule or mass was selected over consolidation. All cases were included in this pulmonary opacities’ analysis regardless of radiographic projection. Pleural effusions were classified according to the extent of lung opacity: small, defined as costophrenic angle blunting or subpulmonary pleural effusion; moderate, involving half or less of the lung field; and large, involving more than half of the lung field. To assign a single finding per case, the more severe side was selected in cases of bilateral pleural effusion. Pneumothoraxes were classified as mild (lung apex above the clavicle), moderate (lung apex below the clavicle), or severe (significant lung collapse). To assign a single finding per case, the more severe side was selected in cases of bilateral pneumothorax. AP radiographs were excluded from the stratified analyses of pleural effusion and pneumothorax because severity assessment is difficult on AP images.

### Reference standard

Ground truth labels for normal findings, pulmonary opacities, pleural effusion, and pneumothorax were established primarily based on the findings observed on CT. If a lesion was detectable on CT but not on CXR, the case was labeled negative. All labels were initially assigned by a radiology resident (6 years of experience) and reviewed by one board-certified radiologist, specializing in thoracic imaging (25 years of experience).

### AI tools

**Fig. 3:**
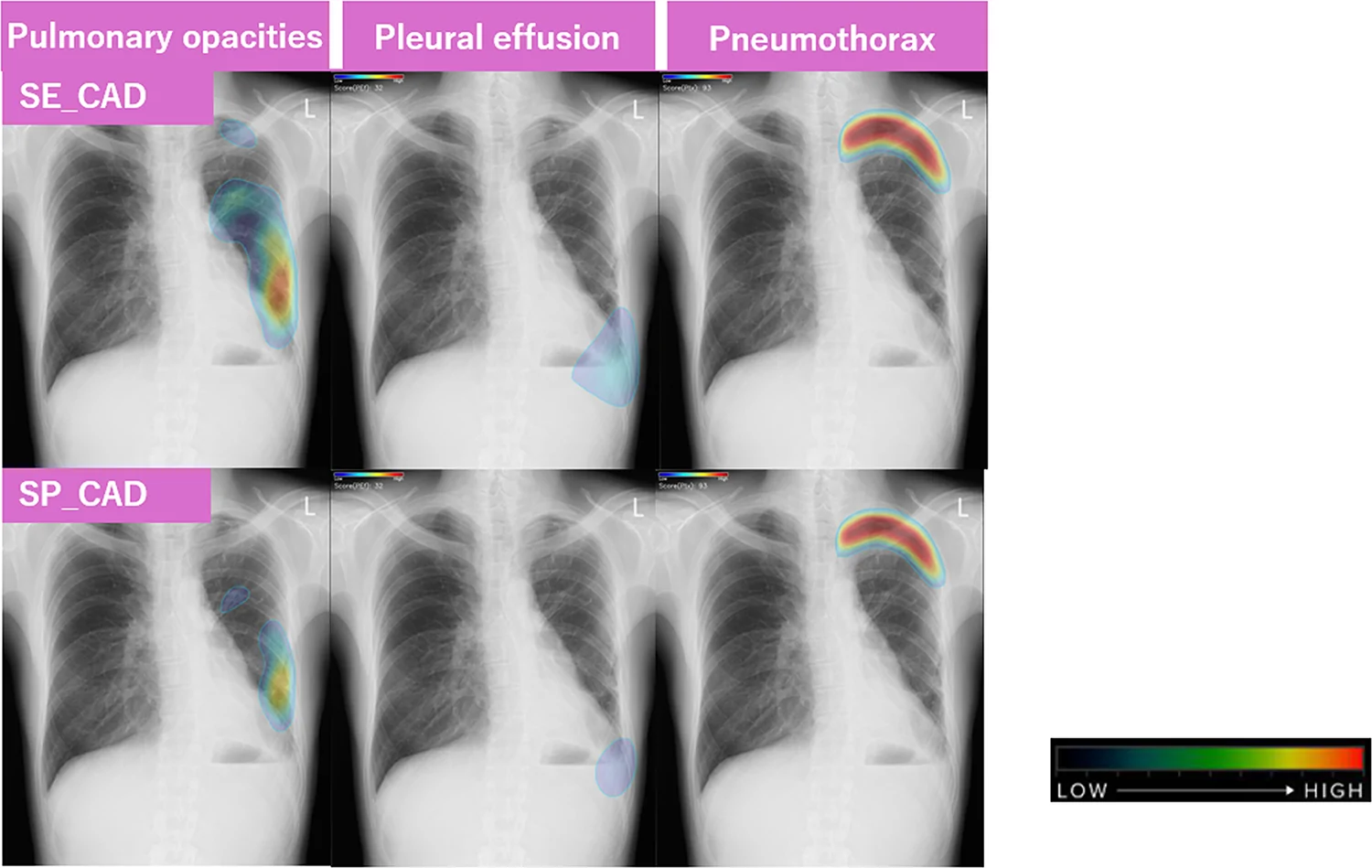
A case of CAD-detected lesions with confidence level visualization. The lesions are color-coded according to the confidence level, with high confidence in red and low confidence in blue on the heatmap SE_CAD = sensitivity-optimized computer aided detection, SP_CAD = specificity-optimized computer aided detection

The AI-based CAD system used in this study was an in-house research system developed to detect pulmonary opacities (nodules/masses/consolidations), pleural effusion, and pneumothorax on CXR. The sensitivity-optimized and specificity-optimized operating modes evaluated in this study are not commercially available. The system was externally validated using an independent dataset from the A university. The system generated pixel-level heat maps with confidence scores ranging from 0 to 1, highlighting regions where each finding was suspected (Fig. 3). A deep-learning detector was employed for model development. Given the well-documented interobserver variability in CXR interpretation [13, 14], the CAD system was trained using high-quality reference labels derived from CT scans to enhance labeling accuracy. Furthermore, the visibility of pulmonary opacities on CXR was quantified based on CT correlation, allowing higher confidence values to be assigned to more conspicuous findings (Supplementary 1). CXRs from 177 cases collected at the B hospital were annotated by three board-certified radiologists to establish the reference standard. The detection performance of the CAD system for three abnormality categories was evaluated using free-response receiver operating characteristic (FROC) analysis, and two operating thresholds—sensitivity-prioritized and specificity-prioritized—were defined for each abnormality (Supplementary 2). Each threshold corresponded to the confidence score output by the CAD system, and sensitivity and false positives per image (FP/image) were calculated at each confidence score threshold. To avoid unstable operating points with excessive false positives, thresholds below 0.10 were excluded from the FROC analysis. The sensitivity-prioritized threshold was set to 0.10, corresponding to the lowest threshold included in the FROC analysis and yielding the highest sensitivity within the evaluated operating range. The specificity-prioritized threshold was defined as the operating point at which the number of FP/image was reduced by approximately 50% compared with the sensitivity-prioritized setting. Thresholds were evaluated in increments of 0.05, and the closest threshold meeting this criterion was selected. As a result, specificity-prioritized thresholds of 0.35 were used for pulmonary opacities, and 0.25 for pleural effusion and pneumothorax. Thresholds were locked prior to any test-set evaluation, with clinical review by board-certified thoracic radiologists.

### Observer performance test design

**Fig. 4:**
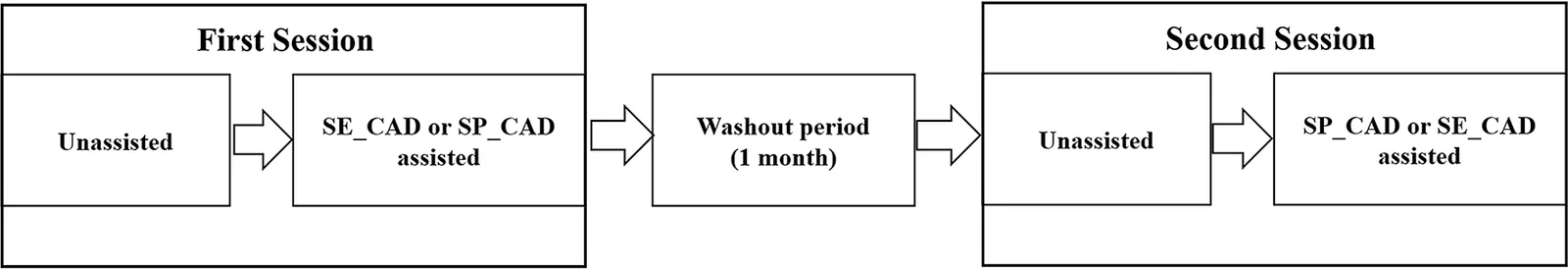
Study reading workflow for radiologist interpretation. All cases were interpreted by each radiologist four times in two sessions. In the first session, all cases were first interpreted without CAD and were then interpreted with either SE_CAD or SP_CAD. After a washout period, a second session was conducted, in which the cases were again interpreted without CAD and were subsequently interpreted with the alternate CAD system that had not been used in the first session. SE_CAD = sensitivity-optimized computer aided detection, SP_CAD = specificity-optimized computer aided detection

A total of six radiologists participated in the observer performance study, including three radiology residents (4, 4 and 5 years of experience) and three board-certified diagnostic radiologists (10, 11 and 23 years of experience). All cases were interpreted by each radiologist a total of four times in two sessions (Fig. 4). In the first session, all cases were initially interpreted without CAD and were then interpreted with either sensitivity-optimized CAD (SE_CAD) or specificity-optimized CAD (SP_CAD). After a one-month washout period, a second session was conducted with the same group of radiologists, in which all cases were again interpreted without CAD and were subsequently interpreted with the alternate CAD system that had not been used in the first session. The order of SE_CAD and SP_CAD was randomized using computer-generated tables, and the readers were blinded to the type of CAD used. Comparative analyses between CAD-assisted and unassisted interpretations were performed within the same session to minimize potential cross-session confounding. Case order was not randomized. In each sitting, readers first interpreted the radiographs without CAD and then re-evaluated the same cases with CAD assistance according to the study design. The potential effects of repeated exposure and reading order were not statistically controlled. Reading time was recorded manually, and CAD was used as a concurrent reader during CAD-assisted interpretation. A total of 9,000 data points were expected based on 375 cases, two reading patterns (SE_CAD/SP_CAD), two reading conditions (unassisted/assisted), and six readers (375 × 2 × 2 × 6). Six data points in the SE-CAD unassisted, SE-CAD assisted, and SP-CAD unassisted groups, and seven data points in the SP-CAD assisted group, were excluded because the recorded end time preceded the start time due to a recording error. Consequently, reading time was analyzed using the remaining 8,975 data points. Image interpretation was performed over multiple sittings rather than in a single day because of the number of cases.

The primary outcomes were: (1) diagnostic performance for detecting the presence or absence of target lesions, and (2) diagnostic performance for detecting both presence and anatomical location by lung zone (upper, middle, lower) and laterality (left, right).

Secondary outcomes included: (1) standalone CAD performance compared to human readers, (2) diagnostic performance comparing AI-assisted versus unassisted reading, (3) comparison between SE_CAD and SP_CAD systems, (4) reading time efficiency with and without CAD assistance, (5) performance differences between expert radiologists and radiology residents, (6) Stratified analyses according to size and severity of findings, and (7) Stratified analyses according to the presence or absence of devices on CXR.

### Statistical analysis

Sensitivity, specificity, positive predictive value, and negative predictive value were calculated for each radiologist, and average values across six participating radiologists were reported for both CAD-assisted and unassisted readings. Area under the curve (AUC) was calculated only for diagnostic performance in detecting the presence or absence of the target lesions. For ROC analysis, radiologists rated their diagnostic confidence using a 10-point ordinal scale, where a score of 10 indicated high confidence in the presence of a target lesion and a score of 1 indicated high confidence in its absence. These confidence scores were used as the decision variable to construct receiver operating characteristic curves on a per-case basis.

For SE_CAD and SP_CAD comparison, sensitivity and specificity were compared using the exact binomial test for proportions, with 95% confidence intervals (CIs) computed using the exact method. Reader-specific and reader-averaged AUC estimates, standard errors, and 95% CIs were obtained using the iMRMC software (Java version v4.0.3; United States), following Gallas et al. [15]. Superiority of CAD-assisted over unassisted reading was concluded when 95% CI for (AUC_assisted − AUC_unassisted) excluded zero positively.

All statistical tests were two-sided (α = 0.05); *p* < 0.05 indicated significance. Analyses were performed using EZR software (Jichi Medical University, Tochigi, Japan, version 1.68) [16], a graphical interface for R (The R Foundation for Statistical Computing, Vienna, Austria).

Reading time was recorded in minutes. The unassisted time ratio was defined as (CAD-assisted time / unassisted time) × 100; values < 100% indicate reduction. Mean difference was defined as SE_CAD minus SP_CAD. P values were calculated using the Wilcoxon signed-rank test for paired comparisons.

## Results

### Standalone CAD performance

Table 2 summarizes unassisted CAD, assisted CAD and standalone CAD performance for the presence detection and presence-plus-location detection. Confusion Matrix values for CAD-unassisted and assisted readings are averaged across readers and reported to one decimal place, whereas standalone CAD values are shown as integers. For presence detection, sensitivity was high across all target findings. For pulmonary opacities, sensitivity of single CAD was 97.1% (SE_CAD) and 92.8% (SP_CAD), specificity was 39.0% (SE_CAD) and 54.7% (SP_CAD) respectively. For pleural effusion, sensitivity was 94.1% (SE_CAD) and 89.9% (SP_CAD), specificity was 90.6% (SE_CAD) and 96.5% (SP_CAD). For pneumothorax, sensitivity was 100% for SE_CAD and SP_CAD, specificity was 98.6% (SE_CAD) and 98.8% (SP_CAD).

**Table 2:** Comparison of Reader Modes with Standalone CAD System for Presence Detection and Presence-plus-Location Detection

Detection Criteria	Findings	CAD	Mode	TP	FN	FP	TN	Se	Sp	PPV	NPV	AUC (95% CI)
Presence	Pulmonary Opacities	SE_CAD	Unassisted	123.3	15.7	106.7	129.3	0.887	0.548	0.536	0.892	0.773 (0.719, 0.826)
			Assisted	127.8	11.2	112.7	123.3	0.920	0.523	0.532	0.917	0.799 (0.745, 0.853)
			Single CAD	135	4	144	92	0.971	0.390	0.484	0.958	0.858 (0.820, 0.896)
		SP_CAD	Unassisted	122.2	16.8	96.5	139.5	0.879	0.591	0.559	0.892	0.788 (0.734, 0.841)
			Assisted	126.7	12.3	100.7	135.3	0.911	0.573	0.557	0.916	0.807 (0.753, 0.861)
			Single CAD	129	10	107	129	0.928	0.547	0.547	0.928	0.849 (0.809, 0.889)
	Pleural Effusion	SE_CAD	Unassisted	101.8	17.2	37.5	218.5	0.856	0.854	0.731	0.927	0.914 (0.889, 0.939)
			Assisted	106.8	12.2	29.3	226.7	0.898	0.885	0.785	0.949	0.945 (0.928, 0.962)
			Single CAD	112	7	24	232	0.941	0.906	0.824	0.971	0.962 (0.938, 0.985)
		SP_CAD	Unassisted	100.5	18.5	39.0	217.0	0.845	0.848	0.720	0.921	0.908 (0.873, 0.944)
			Assisted	104.0	15.0	31.3	224.7	0.874	0.878	0.768	0.937	0.939 (0.913, 0.964)
			Single CAD	107	12	9	247	0.899	0.965	0.922	0.954	0.944 (0.916, 0.972)
	Pneumothorax	SE_CAD	Unassisted	27.0	2.0	1.2	344.8	0.931	0.997	0.959	0.994	0.982 (0.961, 1.000)
			Assisted	27.3	1.7	2.8	343.2	0.943	0.992	0.906	0.995	0.987 (0.980, 0.994)
			Single CAD	29	0	5	341	1.000	0.986	0.853	1.000	0.997 (0.994, 1.000)
		SP_CAD	Unassisted	26.3	2.7	2.0	344.0	0.908	0.994	0.929	0.992	0.975 (0.951, 0.999)
			Assisted	28.3	0.7	2.8	343.2	0.977	0.992	0.909	0.998	0.989 (0.980, 0.998)
			Single CAD	29	0	4	342	1.000	0.988	0.879	1.000	0.997 (0.994, 1.000)
Presence and Location	Pulmonary Opacities	SE_CAD	Unassisted	113.2	25.8	106.7	129.3	0.814	0.548	0.515	0.834	-
			Assisted	118.3	20.7	112.7	123.3	0.851	0.523	0.512	0.856	-
			Single CAD	128	11	144	92	0.921	0.390	0.471	0.893	-
		SP_CAD	Unassisted	112.0	27.0	96.5	139.5	0.806	0.591	0.537	0.838	-
			Assisted	115.5	23.5	100.7	135.3	0.831	0.573	0.534	0.852	-
			Single CAD	113	26	107	129	0.813	0.547	0.514	0.832	-
	Pleural Effusion	SE_CAD	Unassisted	101.5	17.5	37.5	218.5	0.853	0.854	0.730	0.926	-
			Assisted	106.2	12.8	29.3	226.7	0.892	0.885	0.784	0.946	-
			Single CAD	109	10	24	232	0.916	0.906	0.820	0.959	-
		SP_CAD	Unassisted	100.2	18.8	39.0	217.0	0.842	0.848	0.720	0.920	-
			Assisted	103.7	15.3	31.3	224.7	0.871	0.878	0.768	0.936	-
			Single CAD	104	15	9	247	0.874	0.965	0.920	0.943	-
	Pneumothorax	SE_CAD	Unassisted	26.8	2.2	1.2	344.8	0.925	0.997	0.958	0.994	-
			Assisted	27.2	1.8	2.8	343.2	0.937	0.992	0.906	0.995	-
			Single CAD	29	0	5	341	1.000	0.986	0.853	1.000	-
		SP_CAD	Unassisted	26.0	3.0	2.0	344.0	0.897	0.994	0.929	0.991	-
			Assisted	27.8	1.2	2.8	343.2	0.960	0.992	0.908	0.997	-
			Single CAD	29	0	4	342	1.000	0.988	0.879	1.000	-

TP, FN, FP, and TN values for CAD-unassisted and assisted readings are averaged across readers and reported to one decimal place, whereas standalone CAD values are shown as integers. Diagnostic performance is summarized for each finding under five modes: the average of five readers without CAD, the average of six readers with SE_ CAD and SP_CAD, standalone SE_CAD and SP_CAD, based on presence detection and presence-plus-location detection. AUC values are shown with 95% confidence intervals in parentheses

Pulmonary opacities = Nodules, Masses and Consolidations, PPV = positive predictive value, NPV = negative predictive value, TP = true positives, FN = false negatives, FP = false positives, TN = true negatives, Se = Sensitivity, Sp = Specificity, AUC =area under the curve, CI = confidence interval, SE_CAD = sensitivity-optimized CAD, SP_CAD = specificity-optimized CAD, CAD = computer-aided detection

For presence-plus-location detection, sensitivity decreased slightly for all findings compared with presence-only detection. For pulmonary opacities, sensitivity was 92.1% (SE_CAD) and 81.3% (SP_CAD), with specificity 39.0% and 54.7%, respectively. For pleural effusion, sensitivity was 91.6% (SE_CAD) and 87.4% (SP_CAD) with specificity 90.6% and 96.5%, respectively. For pneumothorax, sensitivity remained 100% for both CAD, and specificity was 98.6% (SE_CAD) and 98.8% (SP_CAD).

### Diagnostic performance with and without CAD

Table 3 demonstrates diagnostic performance with and without CAD assistance for presence detection and presence-plus-location detection. For pulmonary opacities, both CAD-assisted readings significantly increased sensitivity compared with unassisted reading for presence detection (both CAD: *p* < 0.001) and presence-plus-location detection (SE_CAD: *p* < 0.001, SP_CAD: *p* = 0.004), while specificity significantly declined for presence detection (SE_CAD: *p* = 0.006, SP_CAD: *p* = 0.03) and presence-plus-location detection (SE_CAD: *p* = 0.006, SP_CAD: *p* = 0.03). For pleural effusion, CAD assistance improved AUC for presence detection (both CAD: *p* = 0.04). For pneumothorax, SE_CAD　assistance showed no significant sensitivity difference (*p* = 0.75) but decreased specificity (*p* = 0.03) than unassisted reading, and SP_CAD-assisted improved sensitivity (*p* = 0.003) but showed no significant specificity difference (*p* = 0.23) for presence detection. Similar trends were observed for presence-plus-location detection.

**Table 3:** Diagnostic Performance of AI-Assisted vs. Unassisted Reading (SE_CAD and SP_CAD) for Presence Detection and Presence-plus-Location Detection

Detection Criteria	Findings	Parameter	SE_CAD			SP_CAD		
			Unassisted	Assisted	*p*	Unassisted	Assisted	*p*
Presence	Pulmonary Opacities	Se	0.887 (740/834)	0.920 (767/834)	< 0.001	0.879 (733/834)	0.911 (760/834)	< 0.001
		Sp	0.548 (776/1416)	0.523 (740/1416)	0.006	0.591 (837/1416)	0.573 (812/1416)	0.03
		AUC	0.773 (0.719, 0.826)	0.799 (0.745, 0.853)	0.008	0.788 (0.734, 0.841)	0.807 (0.753, 0.861)	0.006
	Pleural Effusion	Se	0.856 (611/714)	0.898 (641/714)	< 0.001	0.845 (603/714)	0.874 (624/714)	0.02
		Sp	0.854 (1311/1536)	0.885 (1360/1536)	< 0.001	0.848 (1302/1536)	0.878 (1348/1536)	< 0.001
		AUC	0.914 (0.889, 0.939)	0.945 (0.928, 0.962)	0.04	0.908 (0.873, 0.944)	0.939 (0.913, 0.964)	0.04
	Pneumothorax	Se	0.931 (162/174)	0.943 (164/174)	0.75	0.908 (158/174)	0.977 (170/174)	0.003
		Sp	0.997 (2069/2076)	0.992 (2059/2076)	0.03	0.994 (2064/2076)	0.992 (2059/2076)	0.23
		AUC	0.982 (0.961, 1.000)	0.987 (0.980, 0.994)	0.65	0.975 (0.951, 0.999)	0.989 (0.980, 0.998)	0.27
Presence and Location	Pulmonary Opacities	Se	0.814 (679/834)	0.851 (710/834)	< 0.001	0.806 (672/834)	0.831 (693/834)	0.004
		Sp	0.548 (776/1416)	0.523 (740/1416)	0.006	0.591 (837/1416)	0.573 (812/1416)	0.03
	Pleural Effusion	Se	0.853 (609/714)	0.892 (637/714)	< 0.001	0.842 (601/714)	0.871 (622/714)	0.02
		Sp	0.854 (1311/1536)	0.885 (1360/1536)	< 0.001	0.848 (1302/1536)	0.878 (1348/1536)	< 0.001
	Pneumothorax	Se	0.925 (161/174)	0.937 (163/174)	0.77	0.897 (156/174)	0.960 (167/174)	0.01
		Sp	0.997 (2069/2076)	0.992 (2059/2076)	0.03	0.994 (2064/2076)	0.992 (2059/2076)	0.23

The table summarizes the average diagnostic performance of the six readers with and without CAD for presence detection and presence-plus-location detection of each abnormality (nodules/masses/consolidations, pleural effusion, pneumothorax). Raw counts are provided in parentheses. AUC values are shown with 95% confidence intervals in parentheses. Sensitivity and specificity *p* values were calculated using the exact binomial test. AUC P values were calculated using the approach of Gallas et al. implemented in the iMRMC package. P values for the superiority hypothesis H0: AUCAided – AUCUnaided < = 0 were calculated

Pulmonary opacities = Nodules, Masses and Consolidations, SE_CAD = sensitivity-optimized CAD, SP_CAD = specificity-optimized CAD, Se = Sensitivity, Sp = Specificity, AUC = area under the curve, CAD = computer-aided detection

*P value < 0.05 was considered statistically significant

### Comparison between SE_CAD vs. SP_CAD

Comparison Between SE_CAD and SP_CAD is summarized in Table 4. For pulmonary opacities, specificity was significantly higher with SP_CAD-assisted than with SE_CAD-assisted reading (57.3% vs. 52.3%, *p* < 0.001). No significant differences were observed in sensitivity or AUC between the two modes.

**Table 4:** Comparison of SE_CAD vs. SP_CAD for Presence Detection

Detection Criteria	Findings	Parameter	SE_CAD Assisted	SP_CAD Assisted	*p*
Presence	Pulmonary Opacities	Se	0.920 (767/834)	0.911 (760/834)	0.46
		Sp	0.523 (740/1416)	0.573 (812/1416)	< 0.001
		AUC	0.799 (0.745, 0.853)	0.807 (0.753, 0.861)	0.39
	Pleural Effusion	Se	0.898 (641/714)	0.874 (624/714)	0.06
		Sp	0.885 (1360/1536)	0.878 (1348/1536)	0.36
		AUC	0.945 (0.928, 0.962)	0.939 (0.913, 0.964)	0.65
	Pneumothorax	Se	0.943 (164/174)	0.977 (170/174)	0.18
		Sp	0.992 (2059/2076)	0.992 (2059/2076)	1.00
		AUC	0.987 (0.971, 1.000)	0.989 (0.972, 1.000)	0.82

Diagnostic performance with AI assistance (SE_CAD and SP_CAD) was compared, considering the presence of lesions. Raw counts are provided in parentheses. AUROC values are shown with 95% confidence intervals in parentheses. Sensitivity and specificity P values were calculated using the exact binomial test. AUC P values were calculated using the approach of Gallas et al. implemented in the iMRMC package. P values for the superiority hypothesis H0: AUCAided - AUCUnaided < = 0 were calculated. SE_CAD and SP_CAD denote AI tools optimized for sensitivity and specificity, respectively

Pulmonary opacities = Nodules, Masses and Consolidations, SE_CAD = sensitivity-optimized CAD, SP_CAD = specificity-optimized CAD, Se = Sensitivity, Sp = Specificity, AUC = area under the curve, AI = artificial intelligence, H0 = null hypothesis

*p value < 0.05 was considered statistically significant

### Reading time

Reading time is summarized in Supplementary 3. CAD assistance reduced mean reading time by 41% with SE_CAD and by 46% with SP_CAD. There was no significant difference in reading time between SE_CAD and SP_CAD in the assisted condition (mean difference, − 0.03 ± 0.91; *p* = 0.42).

### Experts vs. non experts performance

Supplementary 4 and 5 demonstrate individual reader performance and experts versus non-experts comparison. For pulmonary opacities, SE_CAD assistance significantly increased sensitivity in non-experts (*p* < 0.001), but not in experts (*p* = 0.12). SP_CAD significantly increased sensitivity in both groups (experts: *p* = 0.002, non-experts: *p* = 0.02). CAD decreased specificity in experts (SE_CAD: *p* = 0.005, SP_CAD: *p* = 0.02), but not in non-experts (SE_CAD: *p* = 0.26, SP_CAD: *p* = 0.45). For pleural effusion, both CAD assistance did not change sensitivity among expert readers (SE_CAD: *p* = 0.07, SP_CAD: *p* = 0.054), whereas specificity increased with both CAD operating modes (SE_CAD: *p* = 0.002, SP_CAD: *p* = 0.006). For pneumothorax, no significant differences were observed in sensitivity, specificity, or AUC between assisted and unassisted readings in either expert or non-expert readers for most comparisons (expect for SP_CAD assistance for non-experts).

### Subgroup analysis

Stratified analyses were performed according to pulmonary opacity subtype (nodule, mass, or consolidation), pleural effusion size, pneumothorax severity, and the presence or absence of medical devices on CXR, which are shown in supplementary 6. For pulmonary opacities, sensitivity was higher for masses than for nodules across unassisted reading (SE_CAD: 95.8 [mass] vs. 87.1% [nodule], SP_CAD: 93.8 vs. 86.1%), CAD-assisted reading (SE_CAD: 95.8 vs. 90.8%, SP_CAD: 94.8 vs. 89.8%), and standalone CAD performance (SE_CAD: 100.0 vs. 95.3%, SP_CAD: 93.8 vs. 89.4%). CAD assistance improved sensitivity for both nodules and masses compared with unassisted reading except for masses under SE_CAD assistance (both unassisted and assisted: 95.8%). Sensitivity for consolidations also improved with CAD assistance (SE_CAD: 89.5 to 93.0%, SP_CAD 89.5 to 92.5%). When stratified by the presence of medical devices, sensitivity for pulmonary opacities was lower in cases with devices than in those without devices for both unassisted (SE_CAD: 78.4 [with] vs. 90.2% [without], SP_CAD: 82.4 vs. 88.7) and CAD-assisted readings (SE_CAD: 87.3 vs. 92.6%, SP_CAD: 86.3 vs. 91.8%). In contrast, standalone CAD showed higher sensitivity in cases with devices (SE_CAD: 100.0 vs. 96.7%, SP_CAD: 94.1 vs. 92.6%). Specificity was consistently lower in cases with devices than in those without devices across unassisted reading (SE_CAD: 45.0 vs. 55.7%, SP_CAD: 46.7 vs. 60.3%), CAD-assisted reading (SE_CAD: 41.7 vs. 53.2%, SP_CAD: 47.5 vs. 58.3%), and standalone CAD performance (SE_CAD: 10.0 vs. 41.7%, SP_CAD: 45.0 vs. 55.6%). For pleural effusion, sensitivity was lower for small effusions than for moderate or large effusions. The improvement in sensitivity associated with CAD assistance was more pronounced for small effusions (for small, SE_CAD: 74.2 [unassisted] to 83.7% [assisted], SP_CAD: 72.2 to 80.7%; for moderate, SE_CAD:96.7 to 96.7%, SP_CAD: 96.4 to 95.5%; for large, SE_CAD: 97.6 to 100.0%, SP_CAD: 100.0 to 100.0%). For pneumothorax, standalone CAD achieved sensitivity of 100.0% across all severity categories. In contrast, sensitivity was lower for mild pneumothorax than for moderate or severe pneumothorax for unassisted (SE_CAD: 75.0 [mild] to 97.1 [moderate] to 100.0% [severe], SP_CAD: 66.7 to 97.1 to 96.7%) and CAD-assisted readings (SE_CAD: 83.3 to 96.1 to 100.0%, SP_CAD: 91.7 to 99.0 to 100.0%). On CXRs, strictly differentiating among ground-glass nodules (GGN), part-solid GGNs, and solid nodules presents significant challenges. Consequently, this study could not conduct a stratified analysis based on the presence or absence of ground-glass components.

## Discussion

This retrospective study demonstrated that CAD improved radiologists’ diagnostic performance for pulmonary opacities, pleural effusion, and pneumothorax on CXRs. Both CAD modes enhanced lesion detection compared with unassisted reading. Notably, for pulmonary opacities, SP_CAD resulted in a significant increase in specificity compared with SE_CAD, while no statistically significant reduction in sensitivity was observed.

A unique strength of this study is the direct comparison of two distinct CAD threshold settings—sensitivity-focused and specificity-focused modes—which suggests that each may differentially influence radiologists’ diagnostic performance. While previous studies have assessed standalone CAD performance under different threshold settings [7–11], these analyses were restricted to CAD-only evaluations. To our knowledge, no prior studies have investigated whether varying threshold settings within the same AI model affect radiologist diagnostic performance under AI assistance.

In our study, only lesions visible on CXRs were considered as ground truth, even though the CAD model was trained using CT-based labels. This design aligns with findings by Kim et al. [17], who showed CAD performance improves when ground truths are based on visible findings on CXRs rather than on CT data. AUC values with standalone CAD were high across all findings, 0.849–0.858 for pulmonary opacities, 0.944–0.962 for pleural effusions, and 0.997 for pneumothoraxes. In addition, standalone CAD demonstrated high sensitivity across all three target conditions. Specificity, however, varied: highest for pneumothorax (98.6–98.8%), moderate for pleural effusion (90.6–96.5%), and lowest for pulmonary opacities (39.0–54.7%). This distribution is similar to the pattern observed among radiologists. Even without CAD, specificity for pulmonary opacities was generally lower than for other conditions, reflecting the diagnostic difficulty of CXRs for pulmonary nodules/masses, in which radiologists may favor sensitivity over specificity to avoid missing clinically relevant lesions. Consistent with the performance pattern observed among radiologists, the CAD system, although trained on CT-based data, also demonstrated lower diagnostic performance for pulmonary opacities than for other findings. Although CXR offers higher in-plane spatial resolution than CT, this does not translate into superior lesion detectability. The lower diagnostic performance of pulmonary opacities on CXR is mainly attributable to the intrinsic limitations of projection imaging, including anatomical superimposition and reduced lesion conspicuity, rather than to spatial resolution itself. Consistent with previous studies [3], pleural effusions and pneumothoraxes demonstrated higher sensitivity and specificity than pulmonary opacities. However, the relatively small number of pneumothorax cases may have influenced the performance estimates, and these results should be interpreted with caution.

Compared with unassisted, CAD-assisted reading for pulmonary nodules was associated with higher sensitivity and AUC but lower specificity. This finding is consistent with several previous studies [3]. CAD systems are often optimized for high sensitivity to minimize missed nodules, which may lead radiologists to classify suspicious findings as positive when CAD marks are present, increasing sensitivity at the expense of specificity. For pleural effusion, CAD-assisted reading showed higher sensitivity, specificity, and AUC than unassisted reading, suggesting a beneficial effect of CAD assistance on diagnostic performance. In contrast, little change in diagnostic performance was observed with CAD assistance for pneumothorax. This may reflect the limited number of pneumothorax cases and the already high baseline performance of radiologists.

When evaluating both presence and location of lesions, specificity remained unchanged while sensitivity slightly declined compared to assessments based solely on presence for CAD unassisted, CAD assisted and single CAD. One possible explanation for the lower sensitivity with localization is the difficulty of defining lesion location. Lesions near zone boundaries may be classified differently by readers, reducing localization-based sensitivity.

This study suggests that different predefined CAD operating points may influence radiologists’ diagnostic performance in CXR interpretation. The findings highlight the importance of operating point selection, as it may affect the balance between sensitivity and specificity. The aim of this study was not to determine a universally optimal operating mode, but to examine whether different predefined operating settings within the same CAD framework influence reader performance. Our findings suggest that reader performance in AI-assisted interpretation may vary according to the selected operating setting, indicating that operating-point design is an important factor in CAD-assisted CXR interpretation.

CAD-assisted readings appeared to reduce interpretation time, however, this finding should be interpreted with caution. Because readers evaluated the same images sequentially without a washout period, the observed reduction is likely to reflect a second-look effect rather than a true improvement in workflow efficiency. Although prior studies, such as Shin HJ, have reported reduced reading times with AI assistance, the present results should be considered reference values rather than definitive evidence of efficiency gains [18]. Comparisons of reading time between SE_CAD and SP_CAD are considered valid, as a washout period was implemented between sessions and readers were blinded to the CAD operating mode. As no significant difference was observed, these findings suggest that the choice of CAD mode did not influence reading time.

In stratified analysis by reader experience, CAD assistance decreased specificity only among experts, whereas non-experts showed no significant change. Experts showed significant sensitivity improvement only with SP_CAD, whereas non-experts demonstrated significant sensitivity improvements with both CAD. This finding consistent with previous studies suggesting that less experienced radiologists tend to derive greater benefit from AI compared with experienced counterparts [19, 20]. Among experts, sensitivity did not differ significantly between unassisted and assisted readings with SE_CAD, whereas SP_CAD significantly improved sensitivity. One possible explanation is that the high number of positive CAD detections may have led radiologists to rely less on AI output. Previous studies have shown that inaccurate AI output can negatively impact radiologists’ judgments [21].

In the stratified analysis according to size and severity of findings, the sensitivity for pulmonary opacities was higher for masses than for nodules across unassisted reading, CAD-assisted reading, and standalone CAD. CAD assistance improved sensitivity for both nodules and masses compared with unassisted reading, except for masses with SE-CAD assistance. Sensitivity for consolidation was also improved with CAD assistance. In case with devices, both unassisted and CAD-assisted readings showed lower sensitivity for pulmonary opacities in cases with devices than in those without devices. In contrast, standalone CAD demonstrated higher sensitivity in cases with devices, suggesting that CAD sensitivity was not adversely affected by devices. Across unassisted reading, CAD-assisted reading, and standalone CAD, specificity was consistently lower in cases with devices than in cases without devices. The presence of medical devices may be associated with more complex radiographic findings, making image interpretation more challenging. For pleural effusion, the improvement in sensitivity with CAD assistance was more pronounced in small-volume effusions. For pneumothorax, standalone CAD achieved 100% sensitivity across all severity categories. In contrast, both unassisted and CAD-assisted readings showed lower sensitivity for mild pneumothorax than for moderate or severe pneumothorax.

This study used a case-enriched dataset to ensure sufficient representation of each abnormality, particularly less frequent findings such as pneumothorax, allowing reliable estimation of diagnostic performance for each condition. Although this design does not reflect real-world disease prevalence and may limit generalizability, it may allow for controlled comparisons of relative differences in diagnostic performance between CAD operating modes.

The concept of adaptive CAD discussed in this study may not be directly applicable to current clinical practice. Freely adjustable operating thresholds could pose regulatory challenges for AI-based software as a medical device (SaMD), as device performance must be validated under controlled conditions. Therefore, the findings of this study should not be interpreted as demonstrating the immediate feasibility of user-adjustable CAD in routine clinical settings, but rather as highlighting the clinical significance of operating point design.

This study has several limitations. First, as a single-center study may limit the generalizability of the findings. Second, variability in lesion annotation between radiologists and the CAD system may have influenced the reference standard and affected performance metrics. Third, the sample size was relatively small, especially for pneumothorax dataset, which may limit generalizability. Fourth, some pneumothorax cases lacked CT images. Although pneumothorax is reliably diagnosed with CXR alone, some cases had additional findings (pulmonary opacities, pleural effusions). These additional findings were included in the analysis despite the absence of CT confirmation, introducing reference standard uncertainty. Fifth, the dataset was artificially enriched with approximately balanced numbers of abnormality categories, and inclusion was limited to patients who underwent CT within 14 days, which does not reflect real-world disease prevalence and may introduce spectrum bias. As a result, the diagnostic performance observed in this study may not directly generalize to clinical settings with lower disease prevalence. Sixth, detailed lesion characteristics, such as nodule size, ground-glass components, pneumothorax severity, and pleural effusion volume, were not systematically analyzed. Because the nodule category in this study included a heterogeneous spectrum of findings, including nodules, masses, and consolidation-type opacities, lesion difficulty could not be fully stratified. Future studies with prospectively defined lesion subcategories are warranted. Seventh, another limitation is the fixed sequential reading design. Because CAD-assisted interpretation was performed following unassisted interpretation without a washout period, the observed results may be influenced by second-look bias, learning effects, or recall rather than the isolated impact of CAD assistance. These factors, alongside potential reader fatigue due to the large case volume, could lead to an overestimation of diagnostic performance and shorter reading times. As these variables were not statistically controlled, they should be carefully considered when interpreting the study’s outcomes. In contrast, comparisons between SE_CAD and SP_CAD are likely more robust, as a washout period was implemented between these sessions, whereas comparisons between CAD-assisted and unassisted readings should be interpreted as exploratory. Finally, our analysis was limited to imaging data. Future studies may benefit from incorporating clinical variables.

In conclusion, different preset operating modes of computer-aided detection significantly influenced diagnostic performance of radiologists in chest radiograph interpretation. A specificity-prioritized setting improved specificity for nodules/masses/consolidations without a statistically significant reduction in sensitivity.

## Supplementary Information

Below is the link to the electronic supplementary material.

Supplementary Material 1

## Data Availability

The data that supports the findings of this study are not openly available due to reasons of sensitivity and are available from the corresponding author upon reasonable request.
