# Supplementary material for: Diagnostic performance with sensitivity/specificity optimized computer-aided detection to detect common abnormalities in chest radiography
Source: Jpn J Radiol. 2026 Aug 3;44(11):1970–81. doi: 10.1007/s11604-026-02053-y (PMC13722758; doi:10.1007/s11604-026-02053-y)
Supplement: Supplementary file 1 [file 11604_2026_2053_MOESM1_ESM.docx]

**Supplementary 1:**

**Confidence Level of AI for Detecting Lung Nodules, Masses and Consolidations**

1.1. **Overview**

The lung nodules, masses and consolidations (pulmonary opacities) detection AI developed in this study is designed to output a confidence level that indicates the visibility of pulmonary opacities. Traditionally, the visibility of pulmonary opacities on chest radiographies (CXRs) has been defined based on the subjective judgement of radiologists [1], but in this study, we attempted to define visibility objectively. When a pulmonary opacity is present on an arbitrary X-ray image, we consider that visibility is high based on the size and density of the corresponding pulmonary opacity on the CT images. The following sections describe the definition of the visibility metric calculated from CT images (1.2), the method for mapping the visibility metric to X-ray images (1.3), and an experiment to evaluate the agreement between the visibility metric and radiologists’ subjective evaluations (1.4).

1.2. **Visibility of Lung Nodules, Masses and Consolidations**

In this study, when CT images are projected into two dimensions based on a projection model of CXR, the X-ray attenuation of the pulmonary opacities region on the two-dimensional X-ray image is calculated on a pixel-by-pixel basis. Simultaneously, the X-ray attenuation in the surrounding region of the pulmonary opacities is also calculated, and the ratio of the two is computed. This is a contrast metric that represents the difference in brightness between the pulmonary opacities area and its surrounding area.

1.3. **Non-Rigid 2D-3D Alignment of Lung Fields**

Position alignment is performed to match the contrast metrics of CXR and CT images. Here, CXR and CT images were taken in different positions and at different times, and the lung field undergoes different deformations due to breathing and other factors in response to rib movement. Non-rigid deformation is necessary for accurate alignment, but since the lung field and ribs overlap in the CXR, it is not possible to align both using normal alignment methods. Therefore, in this study, we first separated the CXR into rib component images and lung field component images [2] and then performed rigid body deformations [3] on the rib parts and non-rigid deformation on the lung field to achieve accurate alignment. For more detailed non-rigid alignment in the latter stage, we optimize the non-rigid transformation that maximizes the similarity between the lung component image separated from the CXR and the image projected onto a two-dimensional plane from the lung area in the CT image. We use B-splines [4] as the basis function for the local non-rigid transformation model, normalized mutual information [5] for similarity, and CMA-ES [6] for optimization.

1.4. **Visibility Evaluation Procedure**

The visibility of pulmonary opacities was evaluated using the following procedure.

1. Extracted cases containing “nodule” or “mass” from radiology reports of chest CT exams of the Department of Radiology at the B hospital.
2. From the extracted cases, collected cases where CXRs were captured within one month of CT scans.
3. Applied pulmonary opacities detection CAD to the CT images and designated the detected regions as pulmonary opacities candidate regions.
4. Two radiologists interpreted the pulmonary opacities candidate regions, and the regions identified as pulmonary opacities by both were designated as pulmonary opacities regions on the CT images.
5. Performed non-rigid 2D-3D alignment between the CT images and CXR images and identified the corresponding positions of the pulmonary opacities regions on the CXRs.
6. Three chest diagnostic specialists evaluated the visibility of rectangular regions containing pulmonary opacities on the CXRs using a six-grade scale (0–5), as defined in the reference [1]. Higher grades indicate greater visibility.
7. Calculated contrast metrics for pulmonary opacities regions on CXRs.
8. Assessed the consistency between the three chest diagnostic specialists’ visibility grades and contrast-derived grades (converted into six levels) using Cohen’s kappa.
   1.5. **Evaluation Results**

The distribution of the average visibility grades by three chest diagnostic specialists and contrast metrics are shown in Figure1-1.

**Figure 1-1 Relationship between Radiologist Visibility Grades and Contrast Metrics**

**The x-axis represents maximum contrast within each bounding box, divided into bins ranging from C ≤ 0.00 to C ≤ 0.89. The y-axis shows the subtlety score assigned by radiologists. Sample sizes for each bin are indicated in parentheses below the x-axis labels (ranging from N = 24 to N = 76).**

Additionally, the results of the consistency among the chest diagnosis specialists and between the specialists and contrast metrics are shown in Table 1-1. Furthermore, the distribution of the consistency between the chest diagnosis specialists and the distribution of the consistency between the average visibility grades and the contrast metrics are shown in Figure 1‑2.

**Table 1-1 Consistency of Grades**

| Combination | Cohen’s Kappa |
| --- | --- |
| Reader#01 vs Reader#02 | 0.268 |
| Reader#02 vs Reader#03 | 0.376 |
| Reader#03 vs Reader#01 | 0.260 |
| Avg. (Reader#01, #02) vs Reader#03 | 0.285 |
| Avg. (Reader#01, #02) vs Contrast | 0.455 |
| Avg. (Reader#02, #03) vs Reader#01 | 0.294 |
| Avg. (Reader#02, #03) vs Contrast | 0.599 |
| Avg. (Reader#03, #01) vs Reader#02 | 0.498 |
| Avg. (Reader#03, #01) vs Contrast | 0.590 |
| Avg. (Reader#01, #02, #03) vs Contrast | 0.633 |

**Inter-rater agreement assessed by Cohen's kappa coefficient. This table shows pairwise comparisons between individual readers (#01, #02, #03), comparisons between averaged reader scores and individual readers, and comparisons between averaged reader scores and contrast-based metrics.**

**Figure 1-2 Distribution of Grade Consistency**

**Heatmaps comparing subtlety score distributions. Left panel: Agreement between reference standard and Reader #3. Right panel: Agreement between reference standard and maximum contrast in a bounding box. Both axes range from 0 to 5, creating a 5×5 grid (25 cells total) for each panel. Cell color intensity represents the frequency of observations for each score combination.**

1.6. **Discussion**

Similar patterns were observed in the distributions of thoracic radiologists’ visibility grades and the contrast metrics. Pairwise correlation coefficients among radiologists were low, indicating weak inter-radiologist agreement. By contrast, the correlations between the radiologists’ mean grades and the contrast metrics were relatively higher, indicating stronger associations than those observed among individual radiologists. These findings suggest that contrast metrics are relatively independent of radiologists’ subjective judgments and can quantify the visibility of pulmonary opacities on CXRs in a more objective, quantitative manner. Leveraging the contrast metric as a gating/triage threshold could restrict AI detections to pulmonary opacities that are radiographically conspicuous to radiologists, thereby potentially improving model specificity.

**References**

1. Shiraishi, Junji, et al. "Development of a digital image database for chest radiographs with and without a lung nodule: receiver operating characteristic analysis of radiologists' detection of pulmonary nodules.", *American Journal of Roentgenology*, 174.1 (2000): 71-74.
2. Shiode, Ryoya, et al. "2D–3D reconstruction of distal forearm bone from actual X-ray images of the wrist using convolutional neural networks." *Scientific Reports*, 11.1 (2021): 1-12.
3. Hiasa, Yuta, et al. "Recovery of 3D rib motion from dynamic chest radiography and CT data using local contrast normalization and articular motion model." *Medical Image Analysis*, 51 (2019): 144-156.
4. Rueckert, Daniel, et al. "Nonrigid registration using free-form deformations: application to breast MR images." *IEEE transactions on medical imaging*, 18.8 (1999): 712-721.
5. Maes, Frederik, et al. "Multimodality image registration by maximization of mutual information." *IEEE transactions on Medical Imaging*, 16.2 (1997): 187-198.
6. Hansen, Nikolaus, Sibylle D. Müller, and Petros Koumoutsakos. "Reducing the time complexity of the derandomized evolution strategy with covariance matrix adaptation (CMA-ES).", *Evolutionary computation*, 11.1 (2003): 1-18.

**Supplementary 2: ROC curves used for determining CAD operating thresholds for three abnormality categories**

Free-response ROC (FROC) curves are shown for (a) nodules/masses/consolidations, (b) pleural effusion, and (c) pneumothorax. Operating thresholds evaluated at 0.05 increments are plotted as red circles along each curve. Thresholds below 0.10 were excluded to avoid unstable operating points with excessive false positives. Each plotted point corresponds to a confidence score output by the CAD system, with sensitivity and false positives per image calculated at each confidence score threshold. These FROC curves were used to define sensitivity-prioritized and specificity-prioritized operating points for each abnormality category.

ROC indicates receiver operating characteristic; FP, false positives.

(a)

| CAD | Mode | Mean | Time reduction (unassisted/assisted) |
| --- | --- | --- | --- |
| SE_CAD | Unassisted | 1.38 | 41 |
|  | Assisted | 0.82 |  |
| SP_CAD | Unassisted | 1.55 | 46 |
|  | Assisted | 0.84 |  |

(b)

| Mean difference (SE_CAD − SP_CAD) | SD | p value |
| --- | --- | --- |
| -0.03 | 0.91 | 0.42 |

**Supplementary 3:** Reading time comparison between unassisted and AI-assisted interpretation for SE_CAD and SP_CAD systems.

(a) Mean reading time (minutes) for unassisted versus AI-assisted reading modes and percentage reduction in reading time with AI assistance compared to unassisted reading are shown.
(b) Data are presented as mean ± standard deviation. The mean difference is defined as SE_CAD minus SP_CAD. P values were calculated using the Wilcoxon signed-rank test. P value < .05 was considered statistically significant.

SE_CAD = sensitivity-optimized computer aided detection, SP_CAD = specificity-optimized computer aided detection, SD = standard deviation.

| Findings | Reader | CAD | Mode | TP | FN | FP | TN | Se | Sp | PPV | NPV |
| --- | --- | --- | --- | --- | --- | --- | --- | --- | --- | --- | --- |
| Pulmonary Opacities | A | SE_CAD | Unassisted | 111 | 28 | 82 | 154 | 0.799 | 0.653 | 0.575 | 0.846 |
|  |  |  | Assisted | 117 | 22 | 102 | 134 | 0.842 | 0.568 | 0.534 | 0.859 |
|  |  | SP_CAD | Unassisted | 119 | 20 | 89 | 147 | 0.856 | 0.623 | 0.572 | 0.880 |
|  |  |  | Assisted | 127 | 12 | 99 | 137 | 0.914 | 0.581 | 0.562 | 0.919 |
|  | B | SE_CAD | Unassisted | 127 | 12 | 114 | 122 | 0.914 | 0.517 | 0.527 | 0.910 |
|  |  |  | Assisted | 131 | 8 | 118 | 118 | 0.942 | 0.500 | 0.526 | 0.937 |
|  |  | SP_CAD | Unassisted | 123 | 16 | 103 | 133 | 0.885 | 0.564 | 0.544 | 0.893 |
|  |  |  | Assisted | 125 | 14 | 107 | 129 | 0.899 | 0.547 | 0.539 | 0.902 |
|  | C | SE_CAD | Unassisted | 131 | 8 | 112 | 124 | 0.942 | 0.525 | 0.539 | 0.939 |
|  |  |  | Assisted | 128 | 11 | 112 | 124 | 0.921 | 0.525 | 0.533 | 0.919 |
|  |  | SP_CAD | Unassisted | 126 | 13 | 99 | 137 | 0.906 | 0.581 | 0.560 | 0.913 |
|  |  |  | Assisted | 129 | 10 | 102 | 134 | 0.928 | 0.568 | 0.558 | 0.931 |
|  | D | SE_CAD | Unassisted | 117 | 22 | 82 | 154 | 0.842 | 0.653 | 0.588 | 0.875 |
|  |  |  | Assisted | 124 | 15 | 75 | 161 | 0.892 | 0.682 | 0.623 | 0.915 |
|  |  | SP_CAD | Unassisted | 120 | 19 | 65 | 171 | 0.863 | 0.725 | 0.649 | 0.900 |
|  |  |  | Assisted | 126 | 13 | 63 | 173 | 0.906 | 0.733 | 0.667 | 0.930 |
|  | E | SE_CAD | Unassisted | 129 | 10 | 137 | 99 | 0.928 | 0.419 | 0.485 | 0.908 |
|  |  |  | Assisted | 134 | 5 | 146 | 90 | 0.964 | 0.381 | 0.479 | 0.947 |
|  |  | SP_CAD | Unassisted | 123 | 16 | 125 | 111 | 0.885 | 0.470 | 0.496 | 0.874 |
|  |  |  | Assisted | 127 | 12 | 130 | 106 | 0.914 | 0.449 | 0.494 | 0.898 |
|  | F | SE_CAD | Unassisted | 125 | 14 | 113 | 123 | 0.899 | 0.521 | 0.525 | 0.898 |
|  |  |  | Assisted | 133 | 6 | 123 | 113 | 0.957 | 0.479 | 0.520 | 0.950 |
|  |  | SP_CAD | Unassisted | 122 | 17 | 98 | 138 | 0.878 | 0.585 | 0.555 | 0.890 |
|  |  |  | Assisted | 126 | 13 | 103 | 133 | 0.906 | 0.564 | 0.550 | 0.911 |
| Pleural Effusion | A | SE_CAD | Unassisted | 98 | 21 | 31 | 225 | 0.824 | 0.879 | 0.760 | 0.915 |
|  |  |  | Assisted | 106 | 13 | 35 | 221 | 0.891 | 0.863 | 0.752 | 0.944 |
|  |  | SP_CAD | Unassisted | 98 | 21 | 27 | 229 | 0.824 | 0.895 | 0.784 | 0.916 |
|  |  |  | Assisted | 104 | 15 | 26 | 230 | 0.874 | 0.898 | 0.800 | 0.939 |
|  | B | SE_CAD | Unassisted | 113 | 6 | 58 | 198 | 0.950 | 0.773 | 0.661 | 0.971 |
|  |  |  | Assisted | 114 | 5 | 59 | 197 | 0.958 | 0.770 | 0.659 | 0.975 |
|  |  | SP_CAD | Unassisted | 109 | 10 | 34 | 222 | 0.916 | 0.867 | 0.762 | 0.957 |
|  |  |  | Assisted | 111 | 8 | 34 | 222 | 0.933 | 0.867 | 0.766 | 0.965 |
|  | C | SE_CAD | Unassisted | 104 | 15 | 48 | 208 | 0.874 | 0.812 | 0.684 | 0.933 |
|  |  |  | Assisted | 105 | 14 | 23 | 233 | 0.882 | 0.910 | 0.820 | 0.943 |
|  |  | SP_CAD | Unassisted | 106 | 13 | 52 | 204 | 0.891 | 0.797 | 0.671 | 0.940 |
|  |  |  | Assisted | 109 | 10 | 39 | 217 | 0.916 | 0.848 | 0.736 | 0.956 |
|  | D | SE_CAD | Unassisted | 88 | 31 | 15 | 241 | 0.739 | 0.941 | 0.854 | 0.886 |
|  |  |  | Assisted | 96 | 23 | 9 | 247 | 0.807 | 0.965 | 0.914 | 0.915 |
|  |  | SP_CAD | Unassisted | 90 | 29 | 13 | 243 | 0.756 | 0.949 | 0.874 | 0.893 |
|  |  |  | Assisted | 92 | 27 | 4 | 252 | 0.773 | 0.984 | 0.958 | 0.903 |
|  | E | SE_CAD | Unassisted | 106 | 13 | 40 | 216 | 0.891 | 0.844 | 0.726 | 0.943 |
|  |  |  | Assisted | 113 | 6 | 32 | 224 | 0.950 | 0.875 | 0.779 | 0.974 |
|  |  | SP_CAD | Unassisted | 109 | 10 | 74 | 182 | 0.916 | 0.711 | 0.596 | 0.948 |
|  |  |  | Assisted | 112 | 7 | 72 | 184 | 0.941 | 0.719 | 0.609 | 0.963 |
|  | F | SE_CAD | Unassisted | 102 | 17 | 33 | 223 | 0.857 | 0.871 | 0.756 | 0.929 |
|  |  |  | Assisted | 107 | 12 | 18 | 238 | 0.899 | 0.930 | 0.856 | 0.952 |
|  |  | SP_CAD | Unassisted | 91 | 28 | 34 | 222 | 0.765 | 0.867 | 0.728 | 0.888 |
|  |  |  | Assisted | 96 | 23 | 13 | 243 | 0.807 | 0.949 | 0.881 | 0.914 |
| Pneumothorax | A | SE_CAD | Unassisted | 27 | 2 | 4 | 342 | 0.931 | 0.988 | 0.871 | 0.994 |
|  |  |  | Assisted | 29 | 0 | 5 | 341 | 1.000 | 0.986 | 0.853 | 1.000 |
|  |  | SP_CAD | Unassisted | 22 | 7 | 3 | 343 | 0.759 | 0.991 | 0.880 | 0.980 |
|  |  |  | Assisted | 27 | 2 | 4 | 342 | 0.931 | 0.988 | 0.871 | 0.994 |
|  | B | SE_CAD | Unassisted | 25 | 4 | 0 | 346 | 0.862 | 1.000 | 1.000 | 0.989 |
|  |  |  | Assisted | 26 | 3 | 1 | 345 | 0.897 | 0.997 | 0.963 | 0.991 |
|  |  | SP_CAD | Unassisted | 28 | 1 | 1 | 345 | 0.966 | 0.997 | 0.966 | 0.997 |
|  |  |  | Assisted | 28 | 1 | 1 | 345 | 0.966 | 0.997 | 0.966 | 0.997 |
|  | C | SE_CAD | Unassisted | 29 | 0 | 1 | 345 | 1.000 | 0.997 | 0.967 | 1.000 |
|  |  |  | Assisted | 27 | 2 | 4 | 342 | 0.931 | 0.988 | 0.871 | 0.994 |
|  |  | SP_CAD | Unassisted | 29 | 0 | 4 | 342 | 1.000 | 0.988 | 0.879 | 1.000 |
|  |  |  | Assisted | 28 | 1 | 3 | 343 | 0.966 | 0.991 | 0.903 | 0.997 |
|  | D | SE_CAD | Unassisted | 28 | 1 | 0 | 346 | 0.966 | 1.000 | 1.000 | 0.997 |
|  |  |  | Assisted | 27 | 2 | 2 | 344 | 0.931 | 0.994 | 0.931 | 0.994 |
|  |  | SP_CAD | Unassisted | 28 | 1 | 1 | 345 | 0.966 | 0.997 | 0.966 | 0.997 |
|  |  |  | Assisted | 29 | 0 | 3 | 343 | 1.000 | 0.991 | 0.906 | 1.000 |
|  | E | SE_CAD | Unassisted | 28 | 1 | 1 | 345 | 0.966 | 0.997 | 0.966 | 0.997 |
|  |  |  | Assisted | 28 | 1 | 2 | 344 | 0.966 | 0.994 | 0.933 | 0.997 |
|  |  | SP_CAD | Unassisted | 27 | 2 | 1 | 345 | 0.931 | 0.997 | 0.964 | 0.994 |
|  |  |  | Assisted | 29 | 0 | 3 | 343 | 1.000 | 0.991 | 0.906 | 1.000 |
|  | F | SE_CAD | Unassisted | 25 | 4 | 1 | 345 | 0.862 | 0.997 | 0.962 | 0.989 |
|  |  |  | Assisted | 27 | 2 | 3 | 343 | 0.931 | 0.991 | 0.900 | 0.994 |
|  |  | SP_CAD | Unassisted | 24 | 5 | 2 | 344 | 0.828 | 0.994 | 0.923 | 0.986 |
|  |  |  | Assisted | 29 | 0 | 3 | 343 | 1.000 | 0.991 | 0.906 | 1.000 |

**Supplementary 4: The diagnostic performance of all six readers, with and without CAD assistance, when the presence is considered**

Diagnostic performance is summarized for each finding: the average of six readers (A-C: board-certified diagnostic radiologists, D-F: radiology residents) without CAD and with CAD assistance (SE_CAD and SP_CAD), based on presence detection. TP, FN, FP, and TN values represent averages across readers and were rounded to the nearest integer.

Pulmonary opacities = Nodules, Masses and Consolidations, PPV = positive predictive value, NPV = negative predictive value, TP = true positives, FN = false negatives, FP = false positives, TN = true negatives, Se = Sensitivity, Sp = Specificity, SE_CAD = sensitivity-optimized CAD, SP_CAD = specificity-optimized CAD, CAD = computer-aided detection.

| Findings | Parameter | Experts | | | Non-Experts | | |
| --- | --- | --- | --- | --- | --- | --- | --- |
|  |  | SE_CAD Unassisted | SE_CAD Assisted | *p* | SE_CAD Unassisted | SE_CAD Assisted | *p* |
| Pulmonary Opacities | Se | 0.885 (369/417) | 0.902 (376/417) | 0.12 | 0.890 (371/417) | 0.938 (391/417) | <0.001 |
|  | Sp | 0.565 (400/708) | 0.531 (376/708) | 0.005 | 0.531 (376/708) | 0.514 (364/708) | 0.26 |
|  | AUC | 0.807 (0.747, 0.867) | 0.827 (0.763, 0.890) | 0.14 | 0.738 (0.676, 0.801) | 0.771 (0.698, 0.845) | 0.06 |
| Pleural Effusion | Se | 0.882 (419/476) | 0.910 (430/476) | 0.07 | 0.829 (398/476) | 0.885 (423/476) | 0.002 |
|  | Sp | 0.822 (839/1024) | 0.848 (884/1024) | 0.002 | 0.885 (903/1024) | 0.923 (947/1024) | <0.001 |
|  | AUC | 0.923 (0.899, 0.946) | 0.937 (0.919, 0.954) | 0.13 | 0.905 (0.870, 0.940) | 0.953 (0.938, 0.968) | 0.10 |
| Pneumothorax | Se | 0.931 (81/87) | 0.943 (82/87) | 1.00 | 0.931 (81/87) | 0.943 (82/87) | 1.00 |
|  | Sp | 0.995 (1033/1038) | 0.990 (1028/1038) | 0.18 | 0.998 (1036/1038) | 0.993 (1031/1038) | 0.18 |
|  | AUC | 0.973 (0.952, 0.995) | 0.980 (0.968, 0.992) | 0.75 | 0.976 (0.954, 0.999) | 0.986 (0.974, 0.998) | 0.45 |
|  |  | SP_CAD Unassisted | SP_CAD Assisted | *p* | SP_CAD Unassisted | SP_CAD Assisted | *p* |
| Pulmonary Opacities | Se | 0.882 (488/556) | 0.914 (504/556) | 0.002 | 0.875 (473/556) | 0.909 (489/556) | 0.02 |
|  | Sp | 0.589 (554/944) | 0.565 (534/944) | 0.02 | 0.593 (558/944) | 0.582 (545/944) | 0.45 |
|  | AUC | 0.814 (0.761, 0.866) | 0.835 (0.792, 0.877) | 0.09 | 0.762 (0.678, 0.846) | 0.779 (0.691, 0.867) | 0.006 |
| Pleural Effusion | Se | 0.877 (418/476) | 0.908 (432/476) | 0.054 | 0.812 (381/476) | 0.840 (396/476) | 0.17 |
|  | Sp | 0.853 (859/1024) | 0.871 (886/1024) | 0.006 | 0.842 (869/1024) | 0.884 (922/1024) | <0.001 |
|  | AUC | 0.931 (0.898, 0.963) | 0.954 (0.935, 0.973) | 0.22 | 0.886 (0.840, 0.932) | 0.923 (0.888, 0.959) | 0.22 |
| Pneumothorax | Se | 0.908 (79/87) | 0.954 (83/87) | 0.22 | 0.908 (79/87) | 1.000 (87/87) | <0.001 |
|  | Sp | 0.992 (1030/1038) | 0.992 (1030/1038) | 1.00 | 0.996 (1034/1038) | 0.991 (1029/1038) | 0.13 |
|  | AUC | 0.973 (0.941, 1.000) | 0.980 (0.971, 0.989) | 0.75 | 0.977 (0.942, 1.000) | 0.999 (0.996, 1.000) | 0.23 |

**Supplementary 5: Comparison of the average diagnostic performance of three non-experts and three experts, with and without CAD assistance**

Diagnostic performance of expert radiologists versus radiology residents for CXR interpretation with and without CAD assistance (SE_CAD and SP_CAD). P values compare unassisted versus CAD-assisted performance within each group. Raw counts are provided in parentheses. AUC values are shown with 95% confidence intervals in parentheses. Sensitivity and specificity P values were calculated using the exact binomial test. AUC P values were calculated using the approach of Gallas et al implemented in the iMRMC package. P values for the superiority hypothesis H0: AUCAided - AUCUnaided <= 0 were calculated.

Pulmonary opacities = Nodules, Masses and Consolidations, Se = Sensitivity, Sp = Specificity, AUC =area under the curve , CI = confidence interval, SE_CAD = sensitivity-optimized CAD, SP_CAD = specificity-optimized CAD, CAD = computer-aided detection.

**p* value < .05 was considered statistically significant.

| Findings | Subgroup | CAD | Mode | TP | FN | FP | TN | Se | Sp |
| --- | --- | --- | --- | --- | --- | --- | --- | --- | --- |
| Pulmonary opacities | All | SE_CAD | Unassisted | 123.3 | 15.7 | 107.0 | 129.0 | 0.887 | 0.548 |
|  |  |  | Assisted | 127.8 | 11.2 | 113.0 | 123.0 | 0.920 | 0.523 |
|  |  |  | Single CAD | 135 | 4 | 144 | 92 | 0.971 | 0.390 |
|  |  | SP_CAD | Unassisted | 122.2 | 16.8 | 97.0 | 140.0 | 0.879 | 0.591 |
|  |  |  | Assisted | 126.7 | 12.3 | 101.0 | 135.0 | 0.911 | 0.573 |
|  |  |  | Single CAD | 129 | 10 | 107 | 129 | 0.928 | 0.547 |
|  | Nodule | SE_CAD | Unassisted | 74.0 | 11.0 | － | － | 0.871 | － |
|  |  |  | Assisted | 77.2 | 7.8 | － | － | 0.908 | － |
|  |  |  | Single CAD | 81 | 4 | － | － | 0.953 | － |
|  |  | SP_CAD | Unassisted | 73.2 | 11.8 | － | － | 0.861 | － |
|  |  |  | Assisted | 76.3 | 8.7 | － | － | 0.898 | － |
|  |  |  | Single CAD | 76 | 9 | － | － | 0.894 | － |
|  | Mass | SE_CAD | Unassisted | 15.3 | 0.7 | － | － | 0.958 | － |
|  |  |  | Assisted | 15.3 | 0.7 | － | － | 0.958 | － |
|  |  |  | Single CAD | 16 | 0 | － | － | 1.000 | － |
|  |  | SP_CAD | Unassisted | 15.0 | 1.0 | － | － | 0.938 | － |
|  |  |  | Assisted | 15.2 | 0.8 | － | － | 0.948 | － |
|  |  |  | Single CAD | 15 | 1 | － | － | 0.938 | － |
|  | Consolidation | SE_CAD | Unassisted | 34.0 | 4.0 | － | － | 0.895 | － |
|  |  |  | Assisted | 35.3 | 2.7 | － | － | 0.930 | － |
|  |  |  | Single CAD | 38 | 0 | － | － | 1.000 | － |
|  |  | SP_CAD | Unassisted | 34.0 | 4.0 | － | － | 0.895 | － |
|  |  |  | Assisted | 35.2 | 2.8 | － | － | 0.925 | － |
|  |  |  | Single CAD | 38 | 0 | － | － | 1.000 | － |
|  | With Device | SE_CAD | Unassisted | 13.3 | 3.7 | 11.0 | 9.0 | 0.784 | 0.450 |
|  |  |  | Assisted | 14.8 | 2.2 | 11.7 | 8.3 | 0.873 | 0.417 |
|  |  |  | Single CAD | 17 | 0 | 18 | 2 | 1.000 | 0.100 |
|  |  | SP_CAD | Unassisted | 14.0 | 3.0 | 10.7 | 9.3 | 0.824 | 0.467 |
|  |  |  | Assisted | 14.7 | 2.3 | 10.5 | 9.5 | 0.863 | 0.475 |
|  |  |  | Single CAD | 16 | 1 | 11 | 9 | 0.941 | 0.450 |
|  | Without Device | SE_CAD | Unassisted | 110.0 | 12.0 | 95.7 | 120.3 | 0.902 | 0.557 |
|  |  |  | Assisted | 113.0 | 9.0 | 101.0 | 115.0 | 0.926 | 0.532 |
|  |  |  | Single CAD | 118 | 4 | 126 | 90 | 0.967 | 0.417 |
|  |  | SP_CAD | Unassisted | 108.2 | 13.8 | 85.8 | 130.2 | 0.887 | 0.603 |
|  |  |  | Assisted | 112.0 | 10.0 | 90.2 | 125.8 | 0.918 | 0.583 |
|  |  |  | Single CAD | 113 | 9 | 96 | 120 | 0.926 | 0.556 |
| Pleural Effusion | All | SE_CAD | Unassisted | 97.8 | 15.2 | 36.0 | 215.0 | 0.866 | 0.858 |
|  |  |  | Assisted | 102.8 | 10.2 | 28.0 | 223.0 | 0.910 | 0.889 |
|  |  |  | Single CAD | 108 | 5 | 23 | 228 | 0.956 | 0.908 |
|  |  | SP_CAD | Unassisted | 96.8 | 16.2 | 38.0 | 213.0 | 0.857 | 0.85 |
|  |  |  | Assisted | 100.7 | 12.3 | 30.0 | 221.0 | 0.891 | 0.879 |
|  |  |  | Single CAD | 105 | 8 | 8 | 243 | 0.929 | 0.968 |
|  | Small | SE_CAD | Unassisted | 37.8 | 13.2 | － | － | 0.742 | － |
|  |  |  | Assisted | 42.7 | 8.3 | － | － | 0.837 | － |
|  |  |  | Single CAD | 46 | 5 | － | － | 0.902 | － |
|  |  | SP_CAD | Unassisted | 36.8 | 14.2 | － | － | 0.722 | － |
|  |  |  | Assisted | 41.2 | 9.8 | － | － | 0.807 | － |
|  |  |  | Single CAD | 43 | 8 | － | － | 0.843 | － |
|  | Moderate | SE_CAD | Unassisted | 53.2 | 1.8 | － | － | 0.967 | － |
|  |  |  | Assisted | 53.2 | 1.8 | － | － | 0.967 | － |
|  |  |  | Single CAD | 55 | 0 | － | － | 1.000 | － |
|  |  | SP_CAD | Unassisted | 53.0 | 2.0 | － | － | 0.964 | － |
|  |  |  | Assisted | 53.0 | 3.0 | － | － | 0.955 | － |
|  |  |  | Single CAD | 55 | 0 | － | － | 1.000 | － |
|  | Large | SE_CAD | Unassisted | 6.8 | 0.2 | － | － | 0.976 | － |
|  |  |  | Assisted | 7.0 | 0.0 | － | － | 1.000 | － |
|  |  |  | Single CAD | 7 | 0 | － | － | 1.000 | － |
|  |  | SP_CAD | Unassisted | 7.0 | 0.0 | － | － | 1.000 | － |
|  |  |  | Assisted | 7.0 | 0.0 | － | － | 1.000 | － |
|  |  |  | Single CAD | 7 | 0 | － | － | 1.000 | － |
|  | With Device | SE_CAD | Unassisted | 14.2 | 1.8 | 4.5 | 13.5 | 0.885 | 0.750 |
|  |  |  | Assisted | 15.0 | 1.0 | 3.7 | 14.3 | 0.938 | 0.796 |
|  |  |  | Single CAD | 16 | 0 | 4 | 14 | 1.000 | 0.778 |
|  |  | SP_CAD | Unassisted | 13.3 | 2.7 | 4.5 | 13.5 | 0.833 | 0.750 |
|  |  |  | Assisted | 14.2 | 1.8 | 3.8 | 14.2 | 0.885 | 0.787 |
|  |  |  | Single CAD | 15 | 1 | 1 | 17 | 0.938 | 0.944 |
|  | Without Device | SE_CAD | Unassisted | 83.7 | 13.3 | 31.2 | 201.8 | 0.863 | 0.866 |
|  |  |  | Assisted | 87.8 | 9.2 | 24.2 | 208.8 | 0.905 | 0.896 |
|  |  |  | Single CAD | 92 | 5 | 19 | 214 | 0.948 | 0.918 |
|  |  | SP_CAD | Unassisted | 83.5 | 13.5 | 33.2 | 199.8 | 0.861 | 0.858 |
|  |  |  | Assisted | 86.5 | 10.5 | 26.5 | 206.5 | 0.892 | 0.886 |
|  |  |  | Single CAD | 90 | 7 | 7 | 226 | 0.928 | 0.970 |
| Pneumothorax | All | SE_CAD | Unassisted | 26.0 | 2.0 | 1.0 | 335.0 | 0.929 | 0.997 |
|  |  |  | Assisted | 26.3 | 1.7 | 3.0 | 333.0 | 0.940 | 0.992 |
|  |  |  | Single CAD | 28 | 0 | 5 | 331 | 1.000 | 0.985 |
|  |  | SP_CAD | Unassisted | 25.3 | 2.7 | 2.0 | 334.0 | 0.905 | 0.994 |
|  |  |  | Assisted | 27.3 | 0.7 | 3.0 | 333.0 | 0.976 | 0.992 |
|  |  |  | Single CAD | 28 | 0 | 4 | 332 | 1.000 | 0.988 |
|  | Mild | SE_CAD | Unassisted | 4.5 | 1.5 | － | － | 0.750 | － |
|  |  |  | Assisted | 5.0 | 1.0 | － | － | 0.833 | － |
|  |  |  | Single CAD | 6 | 0 | － | － | 1.000 | － |
|  |  | SP_CAD | Unassisted | 4.0 | 2.0 | － | － | 0.667 | － |
|  |  |  | Assisted | 5.5 | 0.5 | － | － | 0.917 | － |
|  |  |  | Single CAD | 6 | 0 | － | － | 1.000 | － |
|  | Moderate | SE_CAD | Unassisted | 16.5 | 0.5 | － | － | 0.971 | － |
|  |  |  | Assisted | 16.3 | 0.7 | － | － | 0.961 | － |
|  |  |  | Single CAD | 17 | 0 | － | － | 1.000 | － |
|  |  | SP_CAD | Unassisted | 16.5 | 0.5 | － | － | 0.971 | － |
|  |  |  | Assisted | 16.8 | 0.2 | － | － | 0.990 | － |
|  |  |  | Single CAD | 17 | 0 | － | － | 1.000 | － |
|  | Severe | SE_CAD | Unassisted | 5.0 | 0.0 | － | － | 1.000 | － |
|  |  |  | Assisted | 5.0 | 0.0 | － | － | 1.000 | － |
|  |  |  | Single CAD | 5 | 0 | － | － | 1.000 | － |
|  |  | SP_CAD | Unassisted | 4.8 | 0.2 | － | － | 0.967 | － |
|  |  |  | Assisted | 5.0 | 0.0 | － | － | 1.000 | － |
|  |  |  | Single CAD | 5 | 0 | － | － | 1.000 | － |
|  | With Device | SE_CAD | Unassisted | 3.0 | 0.0 | 0.0 | 31.0 | 1.000 | 1.000 |
|  |  |  | Assisted | 3.0 | 0.0 | 1.0 | 30.0 | 1.000 | 0.968 |
|  |  |  | Single CAD | 3 | 0 | 1 | 30 | 1.000 | 0.968 |
|  |  | SP_CAD | Unassisted | 3.0 | 0.0 | 0.3 | 30.7 | 1.000 | 0.989 |
|  |  |  | Assisted | 3.0 | 0.0 | 0.8 | 30.2 | 1.000 | 0.973 |
|  |  |  | Single CAD | 3 | 0 | 1 | 30 | 1.000 | 0.968 |
|  | Without Device | SE_CAD | Unassisted | 23.0 | 2.0 | 1.0 | 304.0 | 0.920 | 0.997 |
|  |  |  | Assisted | 23.3 | 1.7 | 1.8 | 303.2 | 0.933 | 0.994 |
|  |  |  | Single CAD | 25 | 0 | 4 | 301 | 1.000 | 0.987 |
|  |  | SP_CAD | Unassisted | 22.3 | 2.7 | 1.7 | 303.3 | 0.893 | 0.995 |
|  |  |  | Assisted | 24.3 | 0.7 | 2.0 | 303.0 | 0.973 | 0.993 |
|  |  |  | Single CAD | 25 | 0 | 3 | 302 | 1.000 | 0.990 |

**Supplementary 6: Diagnostic performance of CAD-assisted and unassisted reading modes stratified by radiological findings and subgroups.**

TP, FN, FP, and TN values for CAD-unassisted and assisted readings are averaged across readers and reported to one decimal place, whereas standalone CAD values are shown as integers.

Nodule/mass/consolidation: Lesions were categorized into three subgroups for stratified analysis: nodules (less than 3 cm), masses (3 cm or larger), and consolidations. To select a single primary finding per case, the largest lesion was selected when multiple nodules or masses were present. If consolidation coexisted with nodules or masses, the nodule or mass was chosen preferentially over the consolidation.

Pleural effusion: Classified based on the extent of lung opacity: Small (costophrenic angle blunting and/or subpulmonary pleural effusion), Moderate (involving half or less of the lung field), and Large (involving more than half of the lung field). In cases of bilateral pleural effusion, the more severe side was selected.

Pneumothorax: Severity was classified as: Mild (the lung apex is located above the clavicle), Moderate (the lung apex is located below the clavicle), and Severe (significant lung collapse). In cases of bilateral pneumothorax, the more severe side was selected.

Anteroposterior images were excluded from the stratified analyses of pleural effusion and pneumothorax because severity assessment is difficult on anteroposterior radiographs.

Medical devices: Cases were stratified based on the presence or absence of medical devices. Included devices were pacemakers, central venous catheters, implantable ports, nasogastric tubes, gastrostomy tubes, chest tubes, and aortic stents.

Pulmonary opacities = Nodules, Masses and Consolidations, PPV = positive predictive value, NPV = negative predictive value, TP = true positives, FN = false negatives, FP = false positives, TN = true negatives, Se = Sensitivity, Sp = Specificity, AUC = area under the curve , CI = confidence interval, SE_CAD = sensitivity-optimized CAD, SP_CAD = specificity-optimized CAD, CAD = computer-aided detection.
